# Effect of relative GGBS/fly contents and alkaline solution concentration on compressive strength development of geopolymer mortars subjected to sulfuric acid

**DOI:** 10.1038/s41598-022-09682-z

**Published:** 2022-04-04

**Authors:** Osama A. Mohamed, Rania Al Khattab, Waddah Al Hawat

**Affiliations:** College of Engineering, Abu Dhabi University, PO Box 59911, Abu Dhabi, UAE

**Keywords:** Engineering, Materials science

## Abstract

The effect of submerging geopolymer mortar samples in highly acidic solution for 7-, 28-, and 90-days on stability of mass and the development of compressive strength development was assessed experimentally. The mortar binder consisted of GGBS or blends of GGBS and fly ash activated using combinations of NaOH and Na_2_SiO_3_ solutions, and samples were cured in room temperature. It was found that maintaining mortar samples continuously under sulfuric acid doesn’t cause reduction compressive strength or mass from one age to the other, up to 90 days. While decalcification, delaumination, and formation of calcium salts due to sulfate attack may have affected mass and strength, submerging samples under water supported formation of geopolymerization products C-A-S-H and N-A-S-H, and consequently increased the mass and compressive strength of cubic mortar samples with fly ash + GGBS blended binder. The resistance of mortar to sulfuric acid remained consistent when mortars were prepared using GGBS:fly ash ratio of 3:1, equal amounts of GGBS and fly ash, and GGBS as sole binder. When geopolymer mortar samples made with each of the three binders was left exposed to air after casting, compressive strength increased from 7- to 28-days after casting, but at 90-days, all mortar samples experienced decrease in compressive strength relative to the 28-day values. The relatively high content of GGBS (≥ 50%) and absence of curing water in relatively dry conditions caused shrinkage cracking and decrease in compressive strength.

## Introduction

The construction industry in general and concrete infrastructure in particular has a colossal environmental footprint. Nearly 8% of the global emission of CO_2_ has been attributed to the process of producing ordinary Portland cement (OPC)^1^. Alkali-activated binders are promising alternatives to OPC for production of structural concrete. Some of the critical design/evaluation considerations for concrete mixes that use ordinary Portland cement (OPC) or alkali-activated materials as binders is the anticipated ability of the structural system to sustain aggressive environments without appreciable loss in strength in the short or long terms. Studies have shown that concrete may experience substantial decrease in compressive strength when exposed to aggressive environment for long period^2^. The rate of water penetration through the pore system of unsaturated concrete is controlled largely by capillary rise absorption. Transport of deleterious chlorides by water through concrete pore system may cause corrosion of steel reinforcing bars and subsequent deterioration of the load-carrying capacity of the structural system^3^. Fly ash and ground granulated blast furnace slag (GGBS) are examples of supplementary cementitious materials (SCM) are capable of enhancing durability and strength of concrete when used as partial replacements of OPC^4–6^. Similarly, mortar/concrete with alkali-activated fly ash and GGBS binders may offer enhanced fresh properties, significant early age/long-term strength development, and long-term durability. In the present study, these industrial byproducts are evaluated as alkali-activated binders, because they not only represent sustainable alternatives to OPC, but also offer an opportunity for producing durable concrete structural systems.

Concrete and mortar prepared using alkali-activated GGBS as sole binder performed very well in several areas including development of relatively higher compressive strength at early age, good resistance to several types of acids, and promising resistance to carbonation. Some of the shortcomings of using GGBS as only binder is that concrete may exhibit high shrinkage, rapid setting, and lower workability/flowability^7–9^. Combining fly ash with GGBS in carefully balanced proportions could extend setting time and enhance flowability. Fly ash contributes to alkali activated GGBS/fly ash blends by extending setting time of concrete/mortar due to the typical slow dissolution of fly ash^10^.

Developing concrete/mortar using alkali activated fly ash and GGBS as blended binders will generally mitigate the shortcomings of using either of the two aluminosilicates as sole binder, regardless of the ratio of GGBS to fly ash^11^. When it comes to sorptivity, compressive strength development, and resistance to acid attack, two blends of fly ash and GGBS binders offered promising results and deserve further research: (1) equal amounts of fly ash and GGBS, and (2) GGBS-to-fly ash ratio of 3:1^12^.

In OPC-based concrete, acids are recognized as a cause for the loss of strength and mass. When high pH sulfuric acid comes in contact with concrete, it lowers the pH of the naturally alkaline concrete. If the pH of concrete drops below the stability limit of the hydrates, they lose calcium and transform into amorphous hydrogels. The acid attack reaction products include calcium salts of the acid and other hydrogels. Calcium hydroxide decomposition and development of gypsum lead to surface scaling and softening of OPC-based concrete^12^.

Significant degradation in mechanical properties has been reported in the literature when concrete elements are in contact with or immersed in deleterious acid-rich environments, such as sewerage systems, for long period of time^13,14^. Degradation of compressive strength in high acidity environments is often accompanied by reduction in concrete mass, which becomes more significant as the content of OPC binder increases, particularly in the case of sulfuric acid^15^. Ren et al.^16^ reported that mortar prepared using alkali activated GGBS/fly ash as binder, with GGBS:fly ash ratio of 60:40 exhibited better resistance to degradation when immersed in sulfuric acid for 150 days, compared to fly ash: GGBS ratio of 60:40. The investigators attributed the enhanced performance to higher C-A-S-H, a product of GGBS geopolymerization.

Zhang et al.^17^ evaluated performance under sulfuric acid of mortars with alkali activated fly ash/GGBS binders, where fly ash contents are 0%, 30%, 70%, and 100%. The authors reported that 100% fly ash provides mortar samples with the best resistance to corrosion caused by sulfuric acid, compared to mortars using GGBS/fly ash binders with lower percentages of fly ash. The authors also noted that increasing GGBS content densified the pore system before samples were submerged in acid solution, and decreased loss of aluminum (dealuminization) from the gel after exposure to acid. However, that did not enhance corrosion resistance to mortar with higher GGBS contents compared to 100% fly ash, as the investigators reported higher porosity after immersion in sulfuric acid in high GGBS content mortars. Samples prepared with 100% fly ash binder exhibited the highest strength retention after a 28-day period of immersion in sulfuric acid, compared to mortars with higher GGBS content. The investigators found that higher GGBS content densifies the gel and improves retention of Al/Si ratio, which typically decreases after acid attack, compared to 100% fly ash binder.

When mortars prepared using alkali activated fly ash are exposed to 3% sulfuric acid, the formation of the soft and soluble gypsum lowered compressive strength. Siddique and Jang^18^ described the resulting deterioration of mortar pore system as severe, and ascribed it to leaching out of gypsum as well as corrosive action of sulfuric acid.

A study by Bakharev^19^ concluded that mortar that uses alkali activated fly ash as sole binder is vulnerable to damage caused by exposure to sulfuric acid. The investigator reported substantial degradation in mass and compressive strength of mortar samples immersed in 5% sulfuric acid, when class F fly ash mortar is activated using a mixture of sodium hydroxide and potassium hydroxide or using only sodium silicate. Sodium hydroxide activator resulted in far better performing fly ash-based geopolymers under sulfuric acid attack. X-ray diffraction (XRD) studies and scanning electron microscopy (SEM) images showed two possible failure modes: (1) formation of fissures in geopolymer matrix, which occurs in high performing fly ash-based geopolymers, (2) formation of grainy fragile structures and crystallization of zeolites, a failure mode that characterizes lower performing geopolymers.

Gu et al.^20^ reported that mortar with alkali activated class F fly ash binder develops higher strength after immersion for 42 days in sulfuric acid solution with pH = 1.0. However, the mortar samples lost 50% of their initial compressive strength after 492 days of exposure to the acid. Lee and Lee^21^ reported that mortar using alkali activated GGBS/fly ash blended binder experienced C-A-S–H decalcification, when subjected to 10% sulfuric acid. Decalcification of geopolymerization gel contributes to formation of gypsum salts. When the fly ash content is greater than or equal to 50%, a substantial amount of N-A-S-H forms. Investigators reported that N-A-S-H is more resistant to sulfuric acid attack than C-A-S-H^21^. It worthy to note that strength development due to formation of geopolymerization product depends on the relative contents of GGBS and fly ash and solution alkalinity^12^. Therefore, even if N-A-S-H is more resilient to acid attack than C-A-S-H in the same binder composition, when comparing different binder compositions, the ones with higher GGBS-to-fly ash ratio end up developing higher compressive.

Allahverdi and Skvara^22^ evaluated degradation mechanisms of mortar prepared using equal amounts of GGBS and fly ash, and subjected to sulfuric acid. The investigators reported that, when acid concentration is high (pH = 1.0), gypsum will form, deposit within the corroded layer(s), and inhibits complete deterioration. However, when acid concentration is low, and exposure is for limited periods of time (up to 90 days), gypsum will not form. Whether acidity is low or high, acid protons will attack Si–O–Al bond and cause ejection of aluminum from the matrix.

While the pH is an important factor, the type of acid is also critical in evaluating the extent of concrete damage due to exposure or immersion in an acidic solution. Acetic acid, for example, is more damaging to concrete/mortar because of the higher solubility of the products caused by the attack^23^. Acetic acid was found to cause almost complete decalcification of the OPC-based matrix, along with complete dissolution of the hydrated and anhydrous phases^24^. However, when the binder is an alkali activated blend of GGBS and fly ash, concrete/mortar exhibited enhanced resistance to damage caused by organic acids including lactic and acetic acids, compared to OPC binder^25^. The resistance of GGBS/fly ash mortar in contrast with OPC-based mortar is demonstrated by lower mass reduction after submersion in sulfuric acid for 360 days^26^. The poorer resistance of OPC-based concrete/mortar to acid attack compared to those using alkali activated fly ash/GGBS blended binder was attributed to the higher amount of susceptible phases in OPC-based concrete/mortar, such as ettrignite (3CaO·Al_2_O_3_·3CaSO_4_·32H_2_O) and calcium hydroxide.

Water is critical for polymerization of alkali-activated composites as it dissolves and transports silicates and aluminates ions. Water also supports formation of monomers and initiation of the polycondensation process. Substantial amount of mixing water remains enclosed inside the polymeric gel network even after the reaction has taken place. This non-evaporable water supports the development of compressive strength at a later stage by making itself available for further dissolution of unreacted Al^3+^ and Si^4+^ compounds^27^.

Collins and Sanjayan^28^ reported that 100 mm × 200 mm alkali-activated slag concrete cylinders that were bath cured and sealed (w/b ratio = 0.5) developed similar compressive strength over time. On the other hand, after 365 days, samples left exposed in the laboratory at 23 °C and 50% RH experienced 41.4% and 53.5% decrease in compressive strength compared to sealed and water-cured cylinders, respectively. Similarly, cylinders experienced a 17.2% loss in compressive strength when exposed directly to air, compared to sealed or bath cured cylinders. The investigators attributed the loss of compressive strength to microcracking that started to appear on exposed samples during the first 3 days after casting. Mercury Intrusion Porosimetric (MIP) assessment of the cylinders showed that exposed samples developed higher total porosity and coarser pore size distribution, compared to bath and sealed cylinders. For exposed samples, porosity increased with distance from the exterior surface of the samples. The investigators attributed the coarser pore size distribution and higher porosity of exposed samples to shrinkage-induced microcracking.

Wardhono et al.^29^ reported that water-cured mortar samples with alkali activated blend of equal GGBS and class F fly ash contents developed the highest 7-day and 28-day strengths, in contrast with any of the other GGBS-to-fly ash ratios. The GGBS amount in all mixes evaluated by the investigators was equal to or greater than 50% of the total GGBS/fly ash binder content. The solution alkalinity was relatively high, induced by the alkaline activator (NaOH) molarity of 15M. In the presence of curing water, high solution alkalinity enhances dissolution and geopolymerization of fly ash, and together with an equal amount of GGBS, they produce a matrix that is dense and highly cross-linked^12^. After 3-days of water curing, mortar samples with GGBS as sole binder exhibited the highest compressive strength in contrast with all other GGBS/fly ash combinations. At such early age, the dissolution of GGBS typically outpaces fly ash, even in high alkalinity activator solution.

Hu et al.^30^ indicated that steam curing of alkali-activated GGBS/fly ash mortar resulted in slightly lower 28-day compressive strength than similar samples subjected to standard water curing. Steam curing enhances early age strength developments, but this high strength does not continue at later ages.

When fly ash is the sole binder for geopolymer concrete, heating is typically necessary to expedite the geopolymerization process at the early age. However, studies by Ruengsyllapanun et al.^31^ on high calcium fly ash geopolymer mortar showed that ambient curing under water will develop compressive strength that may exceed 25 MPa after 28 days when activated using a mixture of sodium hydroxide and sodium silicate. At a low NaOH molarity of 2M, increasing the activator ratio Na_2_SiO_3_/NaOH from 0 to 0.5 led to significant increase in compressive strength.

Studies by Gao^32^ showed that supplementing alkali-activated GGBS/fly ash binder blends with up to 2% microsilica increases compressive strength and reduces porosity. No further improvement in porosity occurred when more than 2% microsilica is added, and in fact, the compressive strength decreased. The positive effect is due to the fact that microsilica itself hydrates and produces reaction products that fill the voids. Increase in compressive strength of concrete with OPC binder occurs when cement is partially replaced with silica fume, up to an optimum replacement value of 15%, beyond which concrete experiences a degradation in compressive strength^33^.

In alkali-activated mortar/concrete where the binder is a blend of fly ash and GGBS, the relative amounts of the two precursors influences the rate of compressive strength development. Ismail et al.^34^ reported a general decrease in compressive strength of water-cured mortar at the ages of 28-days and 90-days when fly ash content in the GGBS/fly ash blend is increased to 25%, and further decrease in strength when fly ash is increased to 50%. Cylindrical samples (100 × 200 mm) were prepared using a w/b ratio of 0.4, and the GGBS/fly ash binder was activated using a sodium metasilicate solution with 8% concentration by weight of the total binder. When the activator consisted of 8% sodium metasilicate solution, mortar samples with GGBS:fly ash ratio of 3:1 exhibited relatively higher compressive strength at the ages of 28 days and 90 days, compared to the other binder combinations. Similarly, Jang et al.^35^ reported higher early strength development when GGBS content in fly ash/GGBS blends is greater than 70%, however, mortar samples experienced rapid setting and shrinkage cracking, compared to mortar samples with lower GGBS content.

At room temperature and without an alkaline activator, concrete prepared using a binder consisting of a high amount of GGBS blended with a small amount of OPC (GGBS/OPC: 80/20) exhibited a slow development of compressive strength compared to concrete that used OPC as sole binder^36^. This slow compressive strength development is due to the fact that the formation of reaction products by GGBS requires the availability of Ca(OH)_2,_ which is produced by the hydration of OPC. When the amount of OPC in the binder mix is small (20% or less), Ca(OH)_2_ will be insufficient for early age strength development.

Keulen et al.^37^ reported that the 7-day, 28-day, and 56-day strength of concrete prepared with alkali-activated fly ash/GGBS blended binder, increase with the dosage of carboxylate superplasticizer up to a maximum amount. A dosage of 3–4 kg/m^3^ was found optimum for 7-day, 28-day, and 56-day compressive strength.

Uppalapati et al.^38^ demonstrated that increasing Na_2_O content from 1.75 to 2.75% increases the 1-, 3-, 7-, and 28-day compressive strength of GGBS + fly ash (GGBS/fly ash = 80%/20% by volume) mortars, activated using a mixture of sodium silicate and sodium sulfate solutions. Similarly, increasing the curing temperature from 20 to 40 °C also consistently increases the compressive strength at each of the curing days.

The objective of the present study is to assess the effect of curing alkali-activated GGBS/fly ash mortar in highly acidic solution on development of compressive strength, in comparison to strength development when mortar samples are cured by exposure to air in the absence of curing water. The study also evaluated the effects of relative GGBS/fly ash contents in the binder and activator solution alkalinity on strength development.

## Methodology

The experimental program was developed to assess the development of compressive strength and resistance of mortar samples to damage caused by immersion in sulphuric acid solution. The influence of activator solution alkalinity, and contents of fly ash/GGBS in the binder on compressive strength were also taken into consideration. The details of the methodology described in this section are available in the literature^12^.

Blended fly ash and GGBS precursors were activated using a mixture of sodium hydroxide (NaOH) and sodium silicate (Na_2_SiO_3_) solutions. Calculated amounts of NaOH flakes were mixed with clean water to create solutions with four concentrations, 10M, 12M, 14M, and 16M. After mixing, the NaOH solution is left until all heat dissipated, before commercial sodium silicate solution was added to the sodium hydroxide solution. The quantity of sodium silicate was precalculated to create various alkaline activator solutions where the Na_2_SiO_3_/NaOH ratios were 1.5, 2.0, or 2.5. Therefore, each of the four NaOH activator solutions (with molarities of 10M, 12M, 14M, and 16M) was developed in three versions with alkaline activator ratio Na_2_SiO_3_/NaOH equal to 1.5, 2.0, or 2.5. As a result, 12 solutions were designed to activate fly ash and GGBS precursors. Mortar samples were mixed so that the 12 alkaline solutions were used to activate each of the three binder combinations including: (1) 100% GGBS (G100), blended binder with GGBS:fly ash ratio of 3:1 (G75F25), and (2) binder with equal amounts of GGBS and fly ash (G50F50). Therefore, mortars were prepared using a total of 36 mixes to assess the effects of the amounts of fly ash and GGBS in the blended binder, molarity of NaOH, and activator ratio. Prior to preparing mortar samples for either compressive strength or sulfuric acid resistance test, a carboxylic superplasticizer was mixed with the activator solution with adjusted dosage until acceptable flowability with limited or no bleeding is achieved. The final superplasticizer dosage applied to all mixes amounts to 2.5% of the weight of binder. The ratios of sand/binder and liquid/binder were maintained 2.75 and 0.55, respectively, for all mixes.

The binder, sand, and activator solution were mixed continuously for five minutes, and mortar samples were then cast in molds. Molds containing samples were vibrated for better compactness and consolidation.

### Material properties

Table 1 shows the chemical constituents of fly ash and GGBS binders used in the study. In fly ash, the percentage of calcium oxide is *CaO* = 4.294% < 18%, therefore it is compliant with ASTM C618^39^ class F fly ash. Similarly, SiO_2_ + Al_2_O_3_ + Fe_2_O_3_ = 91.03%, which is greater than 50%, therefore, it complies with the same ASTM limits for classes C, F and N. Lastly, the content of sulfur trioxide (SO_3_) in fly ash is 0.068%, which is far less than 4%, therefore, it complies with ASTM C618^39^ requirements for classes C, F, and N. Fly ash and GGBS were provided by RMB Group – Concrete Division, Abu Dhabi, United Arab Emirates. The mineralogical composition is typical of class F fly ash, characterized by crystalline products, such as mullite, magnetite, and quartz^40^.

**Table 1:** Chemical constituents of fly ash and GGBS used in the study.

	CaO	SiO_2_	Al_2_O_3_	SO_3_	Fe_2_O_3_	TiO_2_	K_2_O	MnO	SrO	ZrO_2_	CuO	Cr_2_O_3_	Y_2_O_3_	ZnO
GGBS (%)	59.44	25.68	8.12	2.75	1.499	1.048	0.609	0.562	0.125	0.065	0.031	0.026	0.016	0.013
Fly Ash (%)	4.294	58.158	24.351	0.068	8.517	2.565	1.534	0.094	0.062	0.085	0.037	0.047	0.015	0.033

As described earlier, alkaline activation of the precursors was conducted using combinations of sodium hydroxide (NaOH) and commercial sodium silicates solution (SiO_2_ = 28.8 wt%, Na_2_O = 9.8 wt%, and H_2_O = 61.4 wt%).

### Experimental procedure—compressive strength

The compressive strength was determined in accordance with ASTM C109^41^ on 50 mm cubic samples. Each binder was activated by 12 different alkaline activator solutions as indicated earlier, which gives a total of 36 mortar mixes. Two curing conditions were applied to sets of samples prior to testing: (1) one set was left unsealed under ambient temperature in the laboratory for 1, 7, 28, and 90 days and then tested in compression, and (2) a second set was immersed in 10% sulfuric acid solution until the test day after 7, 28, and 90 days.

### Experimental procedure—measurement of change in mass

Resistance of mortar with alkali activated binders to damage caused by immersion in highly acidic sulfuric acid (pH = 1.0) was assessed by calculating change in mass after immersion in sulfuric acid solution, in accordance with ASTM C267^42^. The details of the experimental procedure outlined in this section were published elsewhere by the investigators^12^. After casting cubic samples (20 mm × 20 mm × 20 mm), they were retained in molds for 24 h. Samples were then demolded, weighed using a 0.01 kg scale, and immersed in 10% sulfuric acid (H_2_SO_4_) solution until the test day. Mortar samples were removed from curing tanks at test age of 7-, 28-, or 90-days. After surface drying, samples were reweighed. The number of samples prepared was sufficient to ensure that reported results were the average of three data points for each parameter, including curing age, activator ratio, binder content, and solution molarity. Each seven days, the sulfuric acid solution was replaced, and samples were visually inspected for signs of color change or other forms of deterioration. The change in sample weight due to immersion in sulfuric acid solution, was determined using Eq. (1):

1 $$ weight\,change\left( \% \right) = \left[ {\left( {W_{after} - W_{before} } \right)/W_{before} } \right] \times 100 $$

where $W_{after}$ = sample weight (g) after immersion for prescribed days. $W_{before}$ = sample weight (g) before immersion in acid solution.

## Results and discussion

### Effect of immersion in sulfuric acid on mortar mass

**Figure 1:**
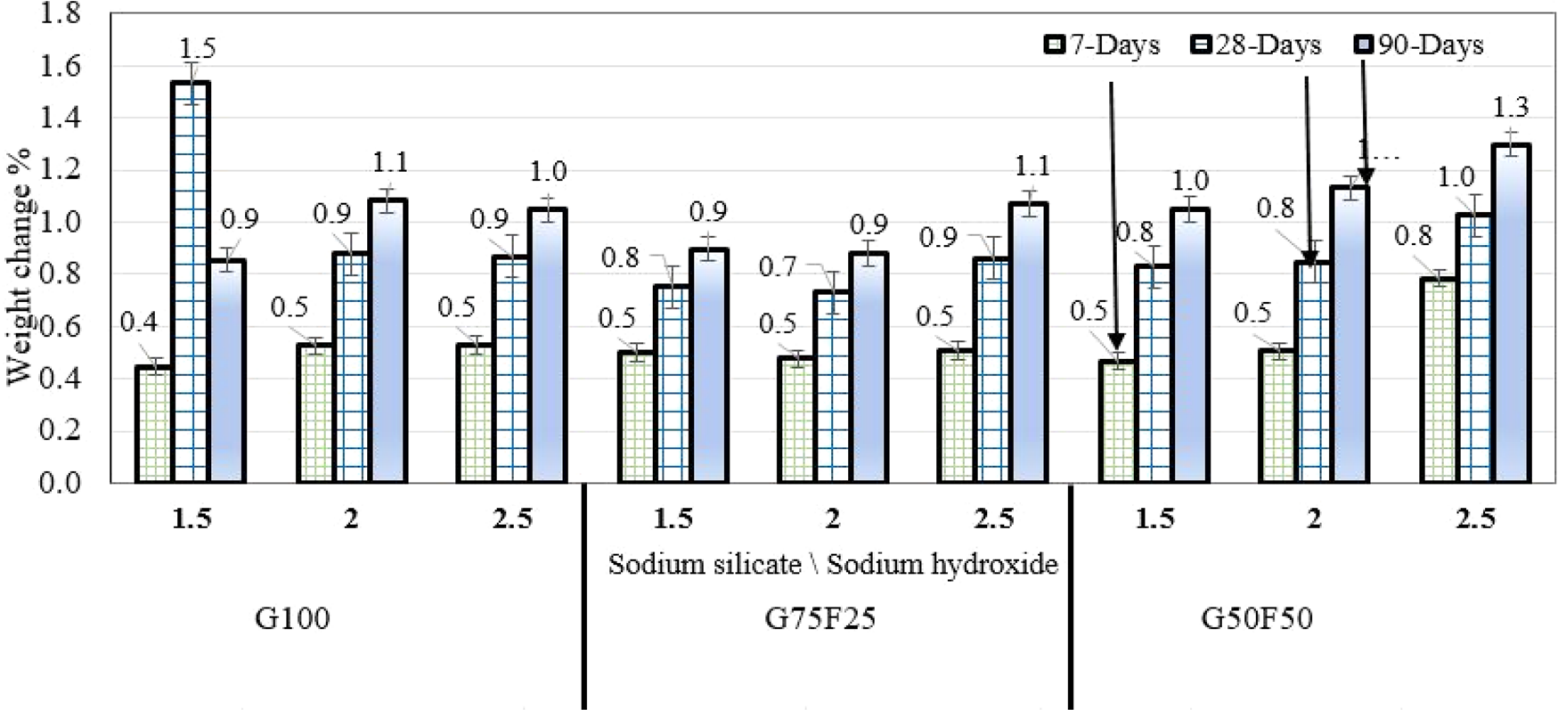
Weight change of G100, G75F25, and G50F50 mortar samples due to immersion in 10% sulfuric acid solution.

The concentration of sulfuric acid in this study is 10%, which makes the curing solution highly acidic. Allahverdi and Skvara^22^ reported that the fundamental corrosion mechanisms of blended GGBS/fly ash mortar in highly acidic sulfuric acid environment include: (1) ejection of aluminum from the aluminosilicate framework, and (2) reaction of calcium ions with sulfate anions to form deposits of gypsum crystals in the corroded layers. The change in mortar weight (%) due to immersion in 10% sulfuric acid for G100, G75F25, and G50F50 samples is shown in Fig. 1. The change in sample weight is positive, indicating increase in weight with curing age for all binder combinations. For mortars prepared with each of the three binder combinations, increasing alkaline activator ratio (1.5, 2.0, 2.5) increases the change in sample weight. Therefore, immersion in highly acidic solution did not cause mass reduction; instead, immersion of samples under water provided a protective medium that contributed to increase in sample weight. This is possibly due to deposition of gypsum and formation of polymerization products that is facilitated by the presence of water. This observation applies to all alkaline activator NaOH concentrations from 10 to 16M. Degirmenci^43^ reported weight loss in GGBS/fly ash mortar samples when they were submerged in sulfuric acid for 24 weeks after casting. In the present study, weight gain can be justified by the deposition of gypsum in highly acidic environment, which played the role of protective layer preventing further deterioration, as hypothesized by Allahverda and Skvara^22^.

Similarly, the sample weight increased with curing age from 7 to 90 days, and the pattern was generally consistent for mortar samples prepared using G100, G75F25, and G50F50. It is worthy to note that when GGBS/fly ash mortars are subjected to low concentration sulfuric acid, deposition of gypsum may not occur and the deterioration mechanism is associated mostly with ejection of aluminum from the aluminosilicate gel, without gypsum deposition^22^. The increase in weight associated with G100 at Na_2_SiO_3_/NaOH ratio of 1.5 is inconsistent with the rest of the data in Fig. 1, and is likely to be an outlier associated with chemical reaction instability or other human factors. The highest increase in weight after 90 days was experienced by G50F50 mortar samples at Na_2_SiO_3_/NaOH ratio of 1.5. G50F50 contains the highest content of fly ash in the present study, which XRD analyses in the literature^27^ showed will experience formation of gypsum (CaSO_4_) and ettrignite (3CaO·Al_2_O_3_·3CaSO_4_·32H_2_O), even at lower acidity level than the higher acidity of 10% in the present study. Gypsum forms due to combination of calcium (Ca^2+^) dissolving from GGBS and fly ash precursors, and SO_4_^2-^ ions from sulfuric acid^17^. In addition to formation of products from sulfate attack, geopolymerization products, such as C-A-S-H and N-A-S-H will also be produced in much higher quantities after 90 days of submersion in water.

### Effect of alkali-activated binder composition on strength development of mortar samples cured in sulfuric acid solution

**Figure 2:**
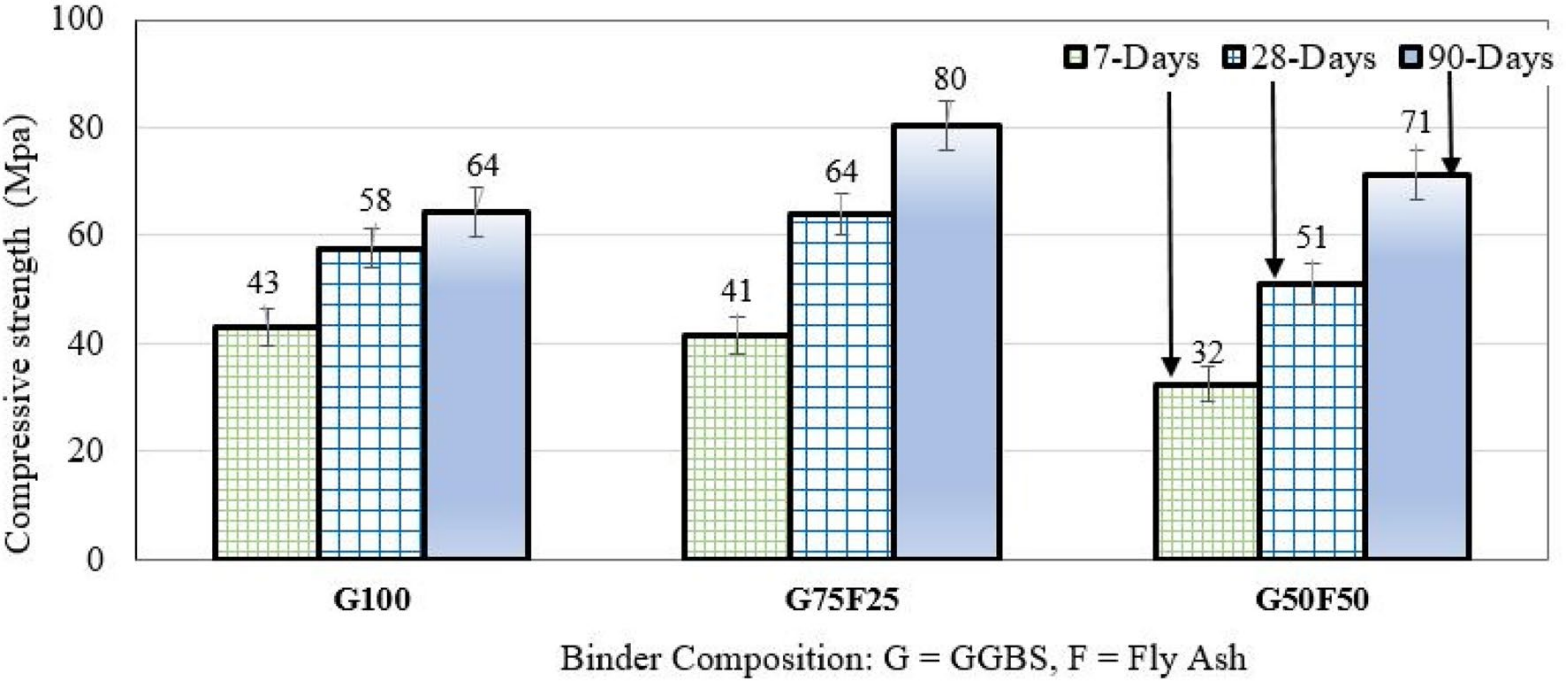
Strength development of G100, G75F25, and G50F50 mortar samples. Data represent the average values for NaOH molarities of 10M, 12M, 14M, and 16M, across activation ratios Na_2_SiO_3_/NaOH = 1.5, 2, and 2.5.

To evaluate the effect of binder composition on compressive strength development, the measured strength was averaged at each curing age across activator concentrations and ratios of sodium hydroxide to sodium solution. Therefore, the observations reported in this section and shown in Fig. 2 apply to mixes prepared with NaOH concentrations from 10 to 16M and Na_2_SiO_3_/NaOH ratios of 1.5, 2.0 and 2.5.

Bakharev et al.^44^ reported that the fundamental deterioration mechanisms due to acid attack on 100%GGBS mortar samples include decalcification of C-S-H and development of soluble salts, yet, no decrease in compressive strength occurred from one test date to another, up to 90 days. Similarly, Fig. 2 shows that compressive strength increased from one curing age to another up to 90 days, not only for G100 but also for G75F25 and G50F50.

Figure 2 also shows that at the age of 7 days, the G100 mortar samples had the highest compressive strength of 43 MPa, while G50F50 developed 32.47 MPa at the same curing age. This is because GGBS reacts faster than fly ash in the early days after casting. After 28 days of curing, G75F25 samples developed higher compressive strength (64.06 MPa) than the G100 mortar samples (57.6 Mpa)). It was hypothesized that the 75% GGBS activates the smaller 25% fly ash, leading to increased compressive strength^12^. This phenomenon continues until the curing age is 90 days, where the compressive strength of G75F25 mortar reaches 80.42 MPa, while G100 mortar develops a strength of 64.44 MPa. Two mechanisms have been hypothesized for the polymerization process of binders containing both GGBS and fly ash, that progress with time: (1) GGBS reacts and forms hydration products, surround the slower dissolving fly ash, and (2) fly ash is activated, produces geopolyermization products, and fills the available pore spaces to provide extra strength^12^. Aiken et al.^45^ reported a decrease in sulfuric acid resistance with increase in GGBS content in alkali-activated GGBS/ash mortars. However, Aiken et al^45^ conducted the test in accordance with ASTM C1898^46^, and samples were cured for 28-days, then immersed in sulfuric acid until compression test was conducted.

The G50F50 mortar samples experienced a relative drop in average compressive strength at the ages of 28- and 90-days, in contrast with G100 and G75F25 samples. This means that the amount of fly ash was too large to be activated by the 50% GGBS even after 90 days of curing. The decrease in compressive strength of the G50F50 mix compared to the G75F25 mix at the age of 28 days is approximately 20.16%. This difference in compressive strength decreases to 11.3% at the age of 90 days, indicating continued activation and polymerization of fly ash.

The higher compressive strength of the G75F25 mortar samples compared to the G100 and G50F50 samples that was observed in the present study was also reported by Ismail^34^.

### Influence of sodium silicate/sodium hydroxide ratio on strength development

The alkaline activator was prepared with NaOH molarities of 10M, 12M, 14M, and 16M. For each molarity, sodium silicate was added so that three mixes were developed with sodium silicate/sodium hydroxide ratios of 1.5, 2.0 and 2.5. The purpose of varying the alkaline activator mix ratio is to investigate its effect on the development of compressive strength, along with the effect of the relative contents of fly ash and GGBS in the binder.

**Figure 3:**
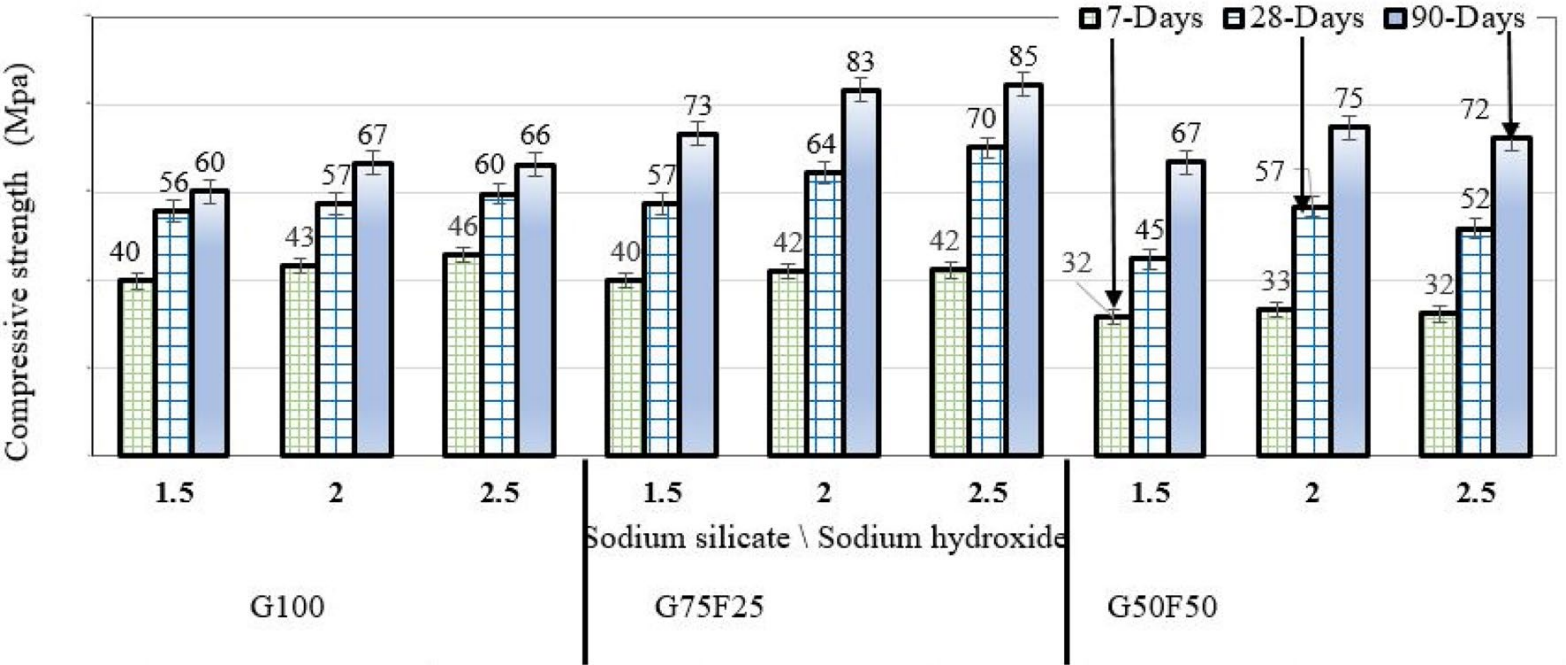
Compressive strength development of G100, G75F25, and G50F50 samples with Na_2_SiO_3_/NaOH ratios of 1.5, 2, and 2.5. Results where the average values of 10M, 12M, 14M, and 16M NaOH mixes.

Figure 3 shows the compressive strength development of 50 mm cubic samples that were submerged in sulfuric acid solution for 7-, 28-, and 90-days for various binder combinations and alkaline activation ratios. Each value of compressive strength for a particular sodium silicate/sodium hydroxide ratio is the average of mixes activated with NaOH with molarities of 10M, 12M, 14M, and 16M. It is clear that increasing the activator ratio (Na_2_SiO_3_/NaOH) generally increases compressive strength for G100, G75F25. However, for G50F50, the activator ratio of 2.0 appears to be the optimum value.

N-A-S-H is the predominant geopolymerization product in systems with limited or no calcium. However, Li et al.^47^ indicated that in calcium-rich systems, similar to the GGBS/fly ash blends in this study, a range of gel structures co-exist including C-S-H, C-A-S-H, N-C-A-S-H, and N-A-S-H. This system is compact and supports strength development*.* Nonetheless, fly ash typically requires longer time to dissolve, and the resulting compact structure need longer curing to manifest itself in the form of increase in compressive strength. For that reason, the 90-day compressive strength was consistently higher in G75F25 and G50F50 than G100 mortars.

### Influence of sodium hydroxide molarity on strength development

**Figure 4:**
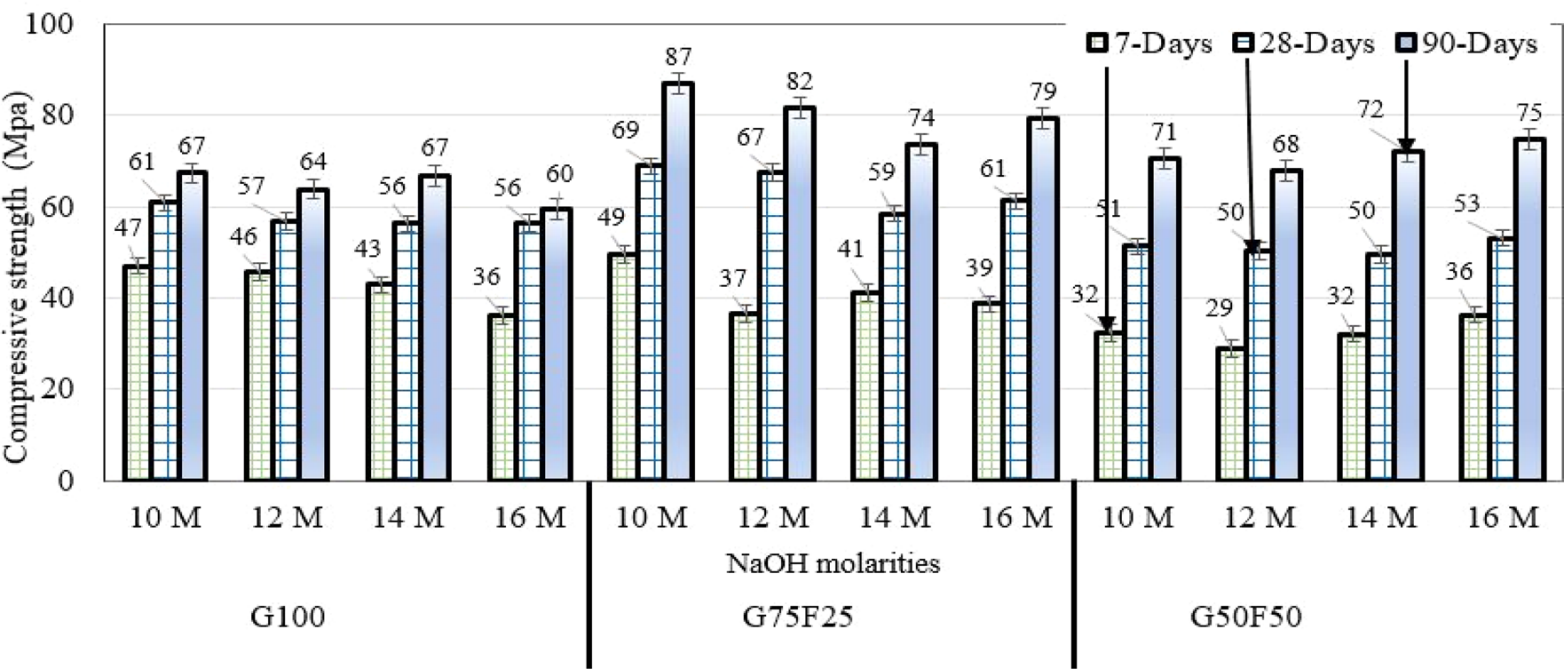
Compressive strength development for water-cured mortar prepared with NaOH molarities of 10M, 12M, 14M, and 16M.

The effect of sodium hydroxide molarity on strength development of water-cured samples is studied by testing 50 mm cubic samples. Figure 4 shows the compressive strength development for mixes prepared with NaOH molarities of 10M, 12M, 14M, and 16M.

Although the mortar samples were cured under 10% sulfuric acid solution, it is clear that all samples increased in compressive strength from the age of 7-days to 28-days, and from 28-days to 90 days. This indicates the geopolymerization products continued to form in the presence of curing water, even in such highly acidic environment. For alkali-activated slag mortars submerged in sodium sulfate solution for one year, XRD analyses published in the literature showed the presence of quartz, C-A-S-H gel, and hydrotalcite, without any other peaks of crystalline products due to sulfate attack^48^.

At the age of 90 days, the G75F25 mortar samples developed the highest compressive strength compared with the G100 and G50F50 samples. The highest compressive strength occurred at the lowest solution alkalinity, when NaOH concentration was 10M. The second highest 90-day compressive strength was developed by G50F50 samples, but at the highest solution alkalinity level, corresponding to NaOH concentration of 16M.

### Effect of curing condition and binder composition on strength development

**Figure 5:**
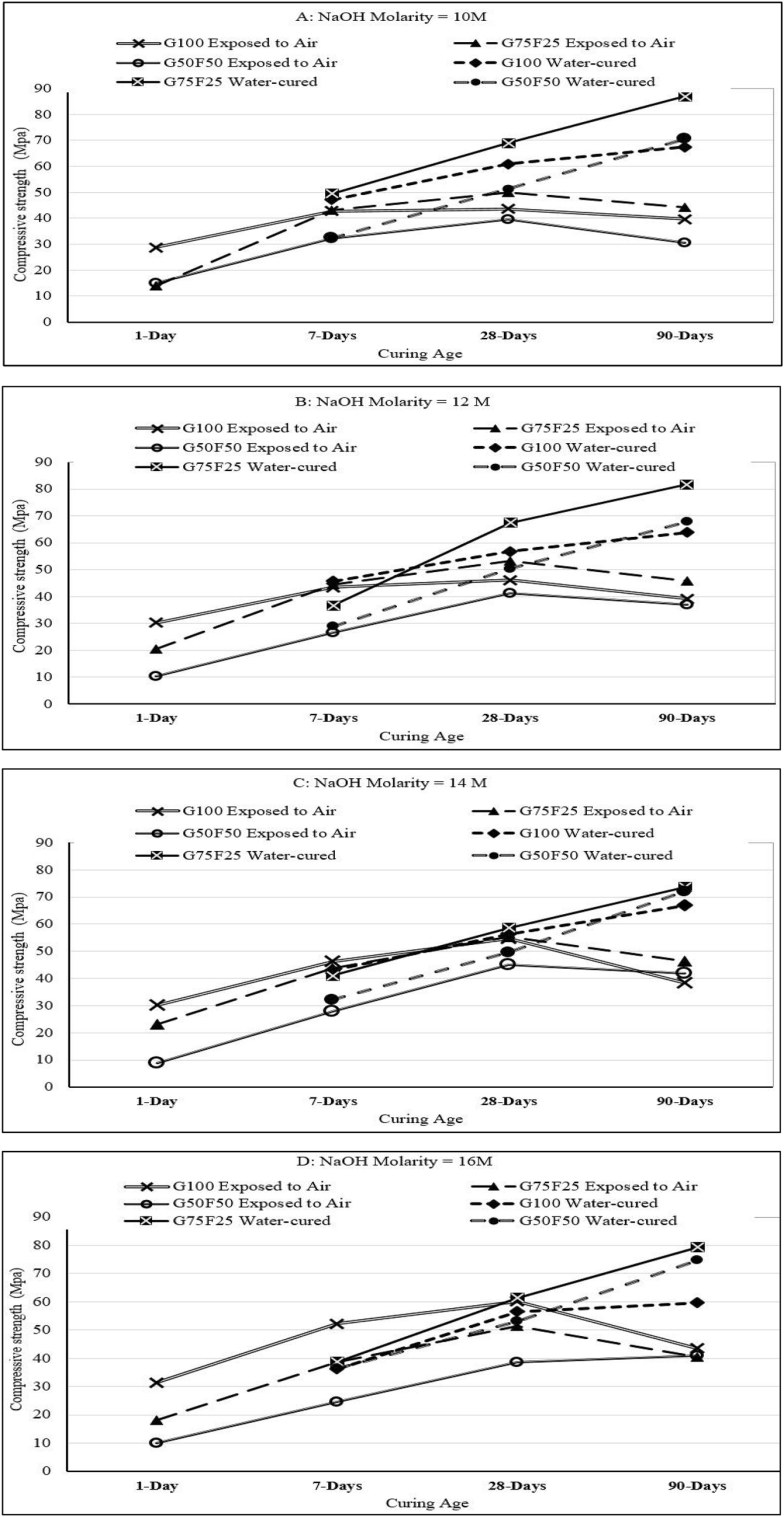
Strength development of G100, G75F25, and G50F50 air-cured and water cured alkali-activated mortars with NaOH molarity of : (**A**) 10M, (**B**) 12M, (**C**) 14M, and (**D**) 16M.

All alkali-activated mortar samples were cured in air under ambient lab conditions after demolding, as described earlier in this article. Another set of samples was cured in 10% sulfuric acid solution to quantify the change in mass and compressive strength due to acid attack. Figure 5 shows the strength development after 7, 28, and 90 days of curing for samples exposed to air in comparison to samples cured in 10% sulfuric acid solution. Figure 5A shows the compressive strength development under each of the two curing conditions when the molarity of NaOH alkaline activator is 10M. Similarly, Fig. 5B represents the strength development when NaOH concentration is 12M, Fig. 5C is for strength development when NaOH molarity is 14M, and Fig. 5D is for mortar activated using NaOH with molarity of 16M. It is clear that mortar samples cured in 10% sulfuric acid solution developed higher strength compared to samples exposed to air. This is consistent with Bernal et al.^49^, who did not observe any decrease in compressive strength of 100% GGBS samples cured in mineral acids, including sulfuric acid, after 150 days of immersion. Samples cured in sulfuric acid solution with pH = 3.0 showed limited drop after 30 days and 60 days, compared to benchmark water-cured samples, but compressive strength exceeded benchmark values after 150 days of curing^49^.

At the early age of 7 days, G100 mortar specimens submersion in sulfuric acid solution developed higher compressive strength for NaOH alkaline activator molarities of 10M, 12M, and 14M, which may be attributed to the faster hydration of GGBS. However, G75F25 and G50F50 had slightly lower compressive strength, as the fly ash dissolved slowly during polymerization by the alkaline activator*.* The reason for the rapid strength development of G100 and to some extent G75F25 is that GGBS is an aluminosilicate where Si–O, Al–O and Ca–O are joined by covalent bonds. The highest bond energy is associated with Si–O, and the lowest is for Ca–O^48^. Therefore, under alkaline activation, breaking of Ca–O bonds occurs first as it requires the least energy, which results in immediate release Ca^2+^ into the mixture. This explains the effect of GGBS on early strength development as the accumulation of Ca^2+^ proceeds to form calcium-rich aluminosilicate gel, along with rapid setting of the mixture. The disjointing of Ca-O is followed by gradual breaking of Al–O and then Si–O bonds.

After 7 days of curing, polymerization of fly ash in G75F25 mortar samples produces more products, while GGBS continues to hydrate and contributes significant amounts of calcium silicate gel until the age of 90 days. This is clearly shown in Fig. 5A–D, where water-cured G75F25 mortar samples exhibited the highest 28-day and 90-day compressive strength in contrast with all other binder compositions. This binder composition developed the highest 90-day compressive strength of 87 MPa in contrast with other binder compositions when the NaOH molarity was 10M. Therefore, increasing fly ash content in G75F25 and G50F50 didn’t cause deterioration due to immersion in highly acidic solution for 7–90 days. Fernandez-Jimenez et al.^50^ `indicated that no significant mineralogical changes appeared in XRD spectra when 100% fly ash mortar samples were submerged in 4.4% sodium sulfate solution for 90 days, and the main reaction product remained aluminosilicate gel.

Figure 5A–D all show a decrease in strength of exposed (air-cured) samples at the age of 90 days, compared to 28-days. This decrease in compressive strength was consistent in all binder combinations (G100, G75F25, and G50F50), and for each NaOH molarity (10M, 12M, 14M, and 16M). A drop in compressive strength of alkali-activated air-cured concrete samples was also reported in the literature for samples tested after curing for 365 days, and was attributed to microcracking^28^. Some studies reported microcracking and decrease in compressive strength due to drying shrinkage when GGBS content was as low as 30% based on tests conducted after 56 days of air curing^9^. Izquierdo et al^51^ attributed the drop in compressive strength of air-cured GGBS/fly ash to the relatively higher porosity leading to leaching of oxyanionic metalloids. In water-cured mortars, the gel is less porous and water represents a physical barrier to leaching of oxyanionic mettaloids, which enhances compressive strength development.

### Effect of alkaline activator ratio on compressive strength development

In this section, the effect of three alkaline activator ratios Na_2_SiO_3_/NaOH = 1.5, 2, and 2.5 on the compressive strength development of mortar samples is reported. The mortar samples tested were G100, G75F25, and G50F50, as described earlier.

**Figure 6:**
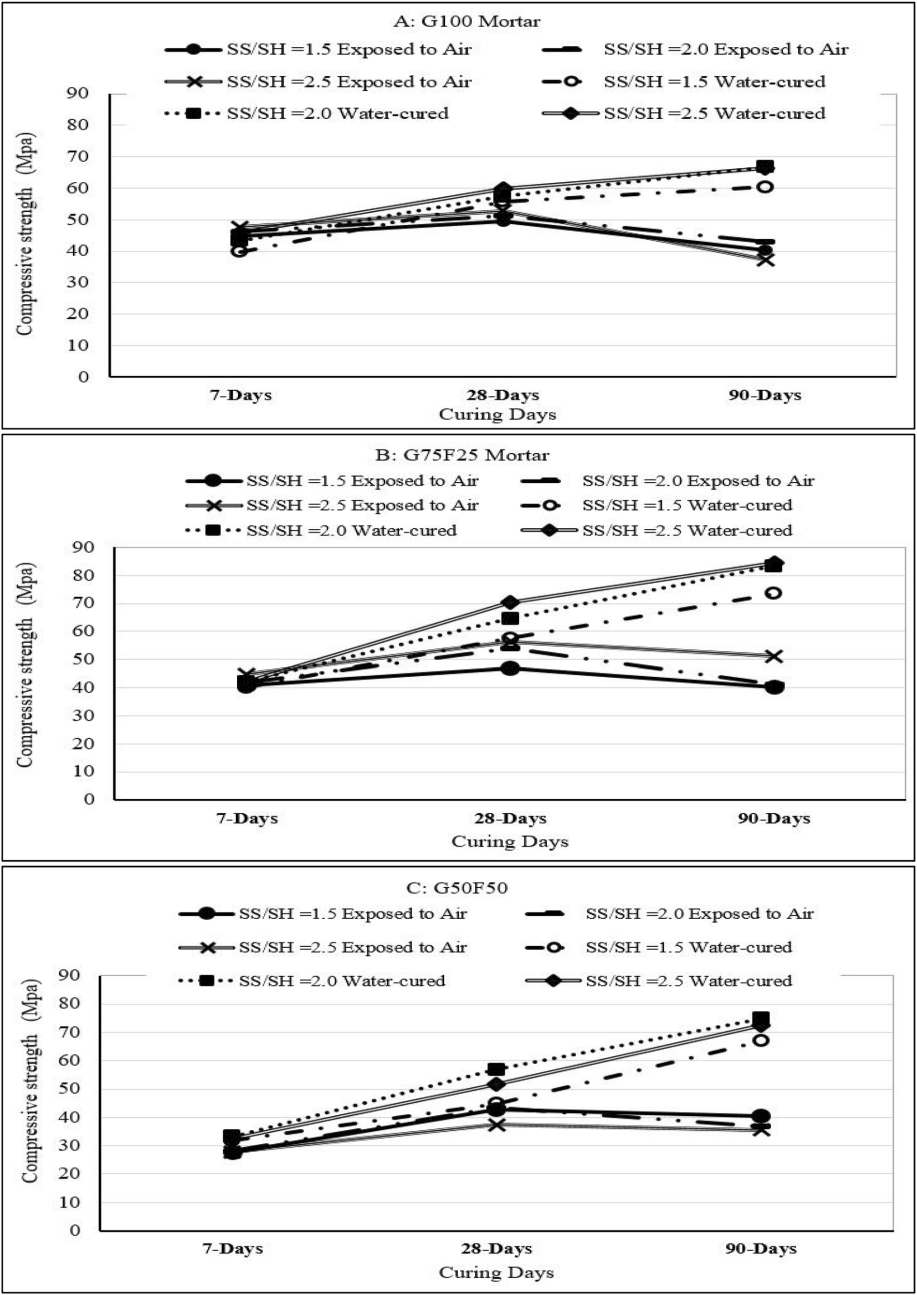
Effect of activator sodium silicate/sodium hydroxide ratio on strength development for mortars with binders consisting of: (**A**) GGBS as sole binder (G100), (**B**) GGBS: fly ash ratio of 3:1 (G75F25), and (**C**) equal amounts of GGBS and fly ash (G50F5).

Figure 6A–C show that the highest 7-day compressive strength of 47.65 MPa was developed by air-cured G100 samples, and 45.83 MPa was developed by water-cured samples, both occurred when Na_2_SiO_3_/NaOH ratio of 2.5. Once GGBS is mixed with the alkaline activator solution, Ca^2+^ debonds quickly from CaO, due to its lower covalent bond energy, and becomes abundantly available^46^ to react with silicates freed from sodium sulfates (Na_2_SiO_3_) and consequently form C-S-H, which is responsible for early strength development. The effect of higher Na_2_SiO_3_/NaOH = 2.5 ratio also persisted with G75F25 samples, where water-cured samples developed the highest 7-day compressive strength of 42.35 MPa and air-cured samples developed 7-day compressive strength of 44.8 MPa, slightly lower than the G100 strengths due to abundance of CaO in G100. For the same reason, the 7-day strength of G50F50 samples (33.2 MPa in the case of water curing and 28.1 MPa for air curing) is by far the lowest of the three binder combinations. In each of the three binders, the difference between the strength of air-cured and water-cured samples was small, largely because at this early age (7 days), loss of moisture from air-cured samples is limited and has insignificant effect on strength development.

After curing for 28-days, G100 samples prepared using the highest Na_2_SiO_3_/NaOH of 2.5 continued to develop higher compressive strength compared to samples with lower Na_2_SiO_3_/NaOH ratios of 1.0 and 2.5, also due to the abundance of Ca^2+^ released from CaO and supply of SiO_3_^2-^, as discussed earlier. At the age of 28 days, the 25% fly ash in G75F25 had long enough time to dissolve, especially in water-cured samples. In GGBS/fly ash blended binders, when GGBS content is greater than or equal to fly ash, the hybrid gel N-C-A-S-H is identified through XRD tests, which is due to the release of calcium by GGBS. The released calcium is then incorporated in the N-A-S-H gel produced by polymerization of fly ash^52^. As result water-cured G75F25 developed the highest 28-day compressive of 70.21 MPa compared to G100 and G50F50. Similarly, in the air-cured set of samples, G75F25 exhibited the highest 28-day compressive of 56.34 MPa compared to G100 and G50F50. Clearly, air-cured samples experienced appreciable decrease in compressive strength compared to water-cured samples, largely due to drying shrinkage. Collins and Sanjayan^28^ reported shrinkage-induced microcracking and decrease in compressive strength in alkali-activated GGBS mortar cured by direct exposure to air.

As shown in Fig. 6, the G75F25 mortar exhibited significantly higher 90-day compressive strength (84.56 MPa), compared to G100 and G50F50, when the activator ratio is Na_2_SiO_3_/NaOH = 2.5. The 90-day strength of air-cured G75F25 is 51.23 MPa, significantly lower than water cured samples. When GGBS content in GGBS/fly ash blended binders is 70% or higher, extensive microcracking of alkali-activated mortar samples was reported in the literature^35^. Figure 5C shows that when the fly ash was further increased to 50% (G50F50), water-cured samples with a Na_2_SiO_3_/NaOH ratios of 2.0 and 2.5 developed statistically similar compressive strength but higher than the strength corresponding to the 1.5 activator ratio. The higher amounts of silicates in the mix provided by higher Na_2_SiO_3_/NaOH ratios contribute to increased formation of calcium silicate by supplementing the silicates dissolving from GGBS, and therefore enhancing development of compressive strength in G100 and G75F25 mortar samples.

## Conclusions

This study evaluated compressive strength development of mortar that uses alkali-activated GGBS/fly ash blended binder cured under 10% sulfuric acid solution, in addition to strength development when mortar samples are cured in air (exposed) at ambient temperature. The effect of relative contents of GGBS and fly ash on mortar response was evaluated by creating three sets of mortars using: (1) GGBS as sole binder (G100), (2) GGBS:fly ash ratio of 3:1 (G75F25), and (3) binder with equal amounts of GGBS and fly ash (G50F50). The alkaline activator solutions were combinations of NaOH and Na_2_SiO_3_. The findings below apply to alkaline activator ratios (Na_2_SiO_3_/NaOH) ranging from 1.5 to 2.5. The effect of activator solution alkalinity on strength development and acid-induced mass reduction was evaluated by creating solutions with NaOH concentration varying from 10 to 16M in increments of 2M.

- Submersion of mortar with alkali-activated binder in sulfuric acid up to 90-days didn’t cause decrease of mass or reduction in compressive strength from one age to another. To the contrary, mass and compressive strength continued to increase indicating formation of geopolymerization products. This doesn’t negate the possibility that the highly acidic environment caused some decalcification, dealumination, or formation/deposition of gypsum salts. However, degradation didn’t prevent growth of mass or compressive strength.
- Mortar samples cured in air at ambient temperature of 22 $\pm$ 2 °C continued to develop compressive strength from until 28-days after demolding as geopolymerization products continued to form in the presence of bound water. However, significant decrease in compressive strength occurred at the age of 90 days compared to the 28-day compressive strength, likely due to loss of moisture leading to shrinkage-induced microcracking.
- Mortar with high GGBS content (greater than or equal to 50%) developed higher compressive strength in low alkalinity activation solution (NaOH molarity = 10M), while mortar with high fly ash content (equal to 50%) developed higher compressive strength in higher alkalinity solution (NaOH molarity = 16M).
